# Hotair promotes the migration and proliferation in ovarian cancer by miR-222-3p/CDK19 axis

**DOI:** 10.1007/s00018-022-04250-0

**Published:** 2022-04-22

**Authors:** Lili Fan, Han Lei, Ying Lin, Zhengwei Zhou, Juanni Li, Anqi Wu, Guang Shu, Sébastien Roger, Gang Yin

**Affiliations:** 1 Department of Pathology, School of Basic Medical Sciences, Xiangya HospitalCentral South UniversityHunan Province, Changsha, 410000 China; 2 Guangzhou Key Laboratory of Formula-Pattern of Traditional Chinese Medicine, School of Traditional Chinese Medicine, Jinan University, Guangzhou, 510632 Guangdong People’s Republic of China; 3 EA4245 Transplantation, Immunologie, Inflammation, University of Tours, 37032 Tours, France

**Keywords:** Ovarian cancer, Hotair/miR-222-3p/CDK19 axis, Proliferation, Migration

## Abstract

Previous studies in our laboratory have reported that miR-222-3p was a tumor-suppressive miRNA in OC. This study aims to further understand the regulatory role of miR-222-3p in OC and provide a new mechanism for its prevention and treatment. We first found that miR-222-3p inhibited the migration and proliferation of OC cells. Then, we observed CDK19 was highly expressed in OC and inversely correlated with miR-222-3p. Besides, we observed that miR-222-3p directly binds to the 3′-UTR of CDK19 and inhibits CDK19 translation, thus inhibiting OC cell migration and proliferation in vitro and repressed tumor growth in vivo. We also observed the inhibitory effect of Hotair on miR-222-3p in OC. In addition, Hotair could promote the proliferation and migration of OC cells in vitro and facilitate the growth and metastasis of tumors in vivo. Moreover, Hotair was positively correlated with CDK19 expression. These results suggest Hotair indirectly up-regulates CDK19 through sponging miR-222-3p, which enhances the malignant behavior of OC. This provides a further understanding of the mechanism of the occurrence and development of OC.

**Supplementary Information:**

The online version contains supplementary material available at 10.1007/s00018-022-04250-0.

## Introduction

The majority of patients with ovarian cancer (OC) lack specific clinical symptoms in the early stages, therefore, usually diagnosed at an advanced stage, and are associated with very pronounced diffuse abdominal metastases, which are more fatal than the primary tumor itself [1–3]. Accordingly, the exploration of key metastasis-driven genes and their underlying mechanisms as well as the identification of effective therapeutic targets are helpful for the development of effective treatment strategies for OC.

It is well established that microRNAs (miRNAs) are usually involved in the post-transcriptional regulation of genes [4]. miRNA plays a key role in many tumor processes, and it can be used as biomarkers for OC diagnosis and prognosis [5]. In our previous study, we reported that miR-222-3p was related to the migration and proliferation of OC [6, 7].

Cyclin-dependent kinase (CDK) is a protein kinase [8]. Selective inhibition of CDK may provide a new treatment for certain cancers [9, 10]. Studies have shown that CDK19 inhibitors exerted antiproliferative activity in prostate cancer cells [11]. Further research indicated that the mediator kinase CDK19 was closely related to tumor migration [12]. However, the roles of CDK19 in OC tumorigenesis have not been reported.

Increasing evidence has revealed that LncRNA is an RNA molecule with a transcription length over 200 nt, which plays a key role in tumor communication [13]. It can participate in a variety of tumor processes, including proliferation, metastasis, angiogenesis, and drug resistance [14, 15]. Hox transcript antisense RNA (Hotair) is the first time to discover trans-acting LncRNA [16]. Hotair regulates multiple functions such as cell cycle, cell migration, and cancer progression [17, 18]. Recent studies have also shown that Hotair is abnormally highly expressed in OC, which is related to proliferation and metastasis in OC cells [19, 20]. But, the mechanisms of Hotair in regulating tumor malignancy are still unclear. Furthermore, the role and potential mechanisms of Hotair in OC remain largely unexplored.

In this study, we investigated the role of Hotair, miR-222-3p, and CDK19 in OC and their relationship. We found that Hotair can promote the proliferation and migration of OC in vitro. Moreover, Hotair indirectly up-regulates CDK19 through sponging miR-222-3p and then influencing the biological behavior of OC. Therefore, our study revealed that Hotair plays an oncogenic role in OC cell, and the Hotair/miR-222-3p/CDK19 axis plays an important role in OC, providing a new rationale for the investigation in the treatment of OC.

## Materials and methods

### Tissue and plasma samples

Tumor tissues along with adjacent healthy tissues of OC patients who underwent tumor resection in the Department of Gynecological Oncology, Hunan Cancer Hospital, Central South University. Informed consent was obtained from all participants before sample collection, and the procedure of tissue collection was approved by the Ethics Review Committee of Xiangya Hospital, Central South University. All experiments strictly adhered to the code of ethics of the World Medical Association and were conducted following the guidelines of Central South University.

### Cell culture and transfection

All cell lines used in this study included the normal ovary cell line IOSE, and OC cell lines, SKOV3, OVCAR3, HO-8910, HO-8910 PM, and A2780 were cultured in RPMI-1640 (BI) replenished with 10% fetal bovine serum (FBS) (BI), 100 µg/mL penicillin (Sigma) and 100 µg/mL streptomycin (Sigma). The human embryonic kidney (HEK)-HEK-293T was cultured in Dulbecco’s Modified Eagle’s Medium (DMEM) (BI) with 10% FBS containing. All cells were cultured at 37 ℃ in a humidified 5% CO_2_ incubator. Cells were collected for the experiment at the indicated time. 5 × 10^5^ OC cells were seeded in a six-well plate and then reached 60–70% confluence per well, lipofectamine 2000 (Thermo Fisher Scientific) was applied for transient transfection overexpression and/or shRNA plasmids according to the manufacturer instructions. These cells were digested and collected after 48 h of transfection for the subsequent assays.

### Bioinformatics analysis

StarBase v2.0 (http://starbase.sysu.edu.cn/) was used to predict potential miRNAs that could be targeted by Hotair. miRPathDB (https://mpd.bioinf.uni-sb.de/), miRDB (http://www.mirdb.org/), miRmap (https://mirmap.ezlab.org/), and StarBase (http://starbase.sysu.edu.cn/) was used to predict potential mRNAs that could be targeted miR-222-3p. TCGA data portal (http://cancergenome.-nih.gov/) was used to analyze the expression levels of Hotair in OC patients. It was using the GTEx (https://www.gtexportal.org/home/index.html) database to show the expression of Hotair in the whole body.

### RNA isolation and qRT-PCR

Total RNA was isolated using TRIzol reagent (Vazyme, Nanjing, China). Complementary DNA (cDNA) was synthesized using the GoScript Reverse Transcription System (Promega, USA). In addition, All-in-One™ miRNA First-Strand cDNA Synthesis Kit (GeneCopoeia, USA, QP013) was used to reverse the transcription of miRNAs. qRT-PCR was performed by the Applied Biosystems 7500 Real-Time PCR System and the GoTaq qRT-PCR Master Mix (Promega, A6001). miRNA was then quantified using the All-in-One™ miRNA qRT-PCR assay kit (GeneCopoeia, USA, QP010). We chose glyceraldehyde-3-phosphate dehydrogenase (GAPDH) to normalize Hotair and CDK19 expression levels and chose U6 as an internal control to normalize miRNA expression levels. Relative RNA abundances were calculated by the standard 2^−ΔΔCt^ method. All the sequences of primers are shown in Supplementary Table S1.

### Western blotting analysis

Cells were lysed with RIPA strong lysis buffer (Beyotime, China) supplemented with the protease inhibitors, 1% PMSF (Roche, Mannheim, Germany), and PIC (Roche, Switzerland). After centrifugation for 15 min, protein concentration in the supernatant was measured using a Pierce™ BCA protein assay kit (Thermo). The supernatant was then mixed with protein loading buffer (NCM Biotech) and boiled at 100 ℃ for 5 min. The same amounts of proteins (30 μg) from all samples were separated by 10% SDS-PAGE gel (Bio-Rad, USA) and transferred onto a PVDF membrane (Immobilon®-P). After blocking in 5% BSA for 1 h at room temperature and incubated overnight at 4 °C with the indicated primary antibodies. Followed by incubation with secondary antibodies for 2 h at room temperature, and visualized by the NcmECL Ultra (A+B) (NCM Biotech) chemiluminescence reagent, GAPDH was used as an internal control. Antibodies against CDK19 were purchased from Proteintech (13761–1-AP, 1:1000) and GAPDH was purchased from Utibody (UM4002, 1:2000). Protein bands were analyzed using Image J software.

### Dual-luciferase reporter assays

The wild-type (wt)/mutant (mut) Hotair or 3′UTR of CDK19 was, respectively, amplified and cloned into plasmids containing luciferase (psiTM-Check2). Then, 5 × 10^4^ HEK-293 T cells were seeded into a 24-well plate. Hotair-wt/mut or CDK19-wt/mut was co-transfected with miR-222-3p mimic/inhibitor or control miRNA into HEK-293T cells with lipofectamine 2000 (Thermo Fisher Scientific). After transfection for 48 h, the luciferase activity was measured with the Dual-Luciferase Reporter Assay System (Promega, USA) according to the manufacturer’s protocol.

### RNA immunoprecipitation (RIP) assay

An RNA Immunoprecipitation (RIP) Kit (Guangzhou, Catalog Bes5101) was used in OC cells to verify the relationship between Hotair and miR-222-3p according to the instructions provided by the manufacturer. Antibodies used for the RIP assay included anti-Argonaute2 (AGO2) and control IgG (BersinBio). Briefly, 1 × 10^7^ cells were lysed in RIP polysome lysis buffer containing protease inhibitor, and RNase inhibitor. Cell lysates were incubated with indicated antibody overnight at 4 °C and then coated with protein A/G beads at 4 °C, 1 h. After extensive washing using RIP wash buffer, the bead-bound immunocomplexes were treated with proteinase K at 55 °C for 1 h. Next, RNA in RIP samples was extracted and the concentration of RNA was measured using a NanoDrop (Thermo Fisher Scientific). Finally, purified RNAs were subjected to qRT-PCR as described above for detection of Hotair and miR-222-3p.

### Colony formation assay

Cells were transfected with different plasmids for 48 h. 500 cells were seeded in a six-well plate and allowed to grow until visible colonies. After 10–14 days, cell colonies were washed with PBS, fixed with 4% paraformaldehyde for 30 min, and stained with crystal violet for 20 min. Image J was used to count the number of colonies per well.

### Flow cytometry assay

Cells were collected and washed with 1 × PBS and resuspended with 1 mL pre-cooled 70% ethanol to fix at 4 ℃ overnight, then transferred and stored at −20 ℃ for later use. After incubation with 300 µL propidium iodide (PI) (BD Biosciences, USA) for 15 min in the dark, the cells were examined on a FACS Calibur system (BD Biosciences, USA) and the results were analyzed by Flowjo.

### EdU assay

Cell-Light EdU DNA Cell Proliferation Kit (RiboBio, Guangzhou, China) was used to perform EdU assay. 3000–5000 cells/well were seeded into a 96-well plate for 12 h. Subsequently, the cells were incubated with 50 µM EdU for 2 h, followed by fixed with 4% paraformaldehyde and stained with Apollo Dye Solution. Finally, the DNAs were stained with Hoechst 33342. A fluorescence microscope (Olympus Corp) was used to obtain images.

### Transwell assay

Cell migration was measured by Transwell assays using the 24-well Transwell chambers with 8 μm polycarbonate membranes (Corning Incorporated, USA). A total of 2 × 10^5^ cells in 200 µL serum-free medium were added to the upper chamber. The lower chamber contained 800 µL medium with 20% FBS as a chemoattractant. After incubation for 20 h, the cells on the lower surface were fixed with 4% paraformaldehyde for 30 min and stained with crystal violet for 20 min. The relative cell number was calculated the light microscope (Olympus, Tokyo, Japan).

### Animal study

Five-week-old female nude mice (BALB/C) were used in all tumor xenograft experiments and purchased from the Laboratory Animal Center of Central South University (Changsha, Hunan, China). We also injected 4 different groups of stable cell lines expressing miR-222-3p and CDK19 into the nude mice at their armpits, as shown in Fig. 3A. Nude mice were euthanized at the end of the experiment (30 days after cell injection), and mice tumors were extracted for RNA and protein. The animal studies were approved by the Laboratory Animal Center of Central South University (Changsha, Hunan, China, the IRB approval number is NO. 2021-KT41).

OC cells (1 × 10^7^ cells in 200 µL PBS) were also stably transfected with Hotair/Vector and suspended with 1 × PBS and injected subcutaneously of the female nude mice (5-week-old, four mice per group, a total of four groups). Tumor growth was examined every 5 days. The mice were killed after 30 days, tumor size and weight were measured. For metastasis experiments, 1 × 10^7^ HO-8910 PM^Vector/or OE−Hotair^ cells in 200 µL PBS were intravenously injected into the intraperitoneal of nude mice (5-week-old, four mice per group, a total of four groups). The experimental treatment was the same as the growth model. 30 days later, mice were euthanized and metastasis nodules were counted.

### Haematoxylin and eosin (HE) staining and immunohistochemistry (IHC)

HE staining was utilized to select representative areas, according to the routine procedure of the department of pathology, Central South University. And the paraffin-embedded sections of OC and normal ovaries were also obtained from the department of pathology, Central South University. The expression levels of CDK19 in ovarian tumors/normal ovarian tissues and the mice’ section of cancerous tissue were evaluated by IHC using an anti-CDK19 antibody (Proteintech, 13761–1-AP, 1:1000). IHC staining was performed according to our previous study [7].

### Statistical analysis

Survival curves were analyzed with the Log-Rank test using the Kaplan–Meier method. The correlation between Hotair, miR-222-3p, and CDK19 expression was determined by calculating Pearson’s correlation coefficient. The unpaired two-tailed t-test was used to analyze the statistical differences (*p*-values) between the two groups. One-way analysis of variance ANOVA followed by Dunnett’s post hoc test was used for multiple comparisons. For quantitative data, all results were collected and expressed as average values ± SD in different assays. All experiments were repeated in triplicate. All statistical analyses were performed using GraphPad Prism 8 software (GraphPad Software, USA). *p* < 0.05 indicated a statistically significant difference. *p < 0.05, **p < 0.01, ***p < 0.001, ****p < 0.0001, revealed by unpaired two-tailed t-test.

## Results

### miR-222-3p was significantly down-regulated in OC and inhibited cell proliferation and migration in vitro

**Fig. 1:**
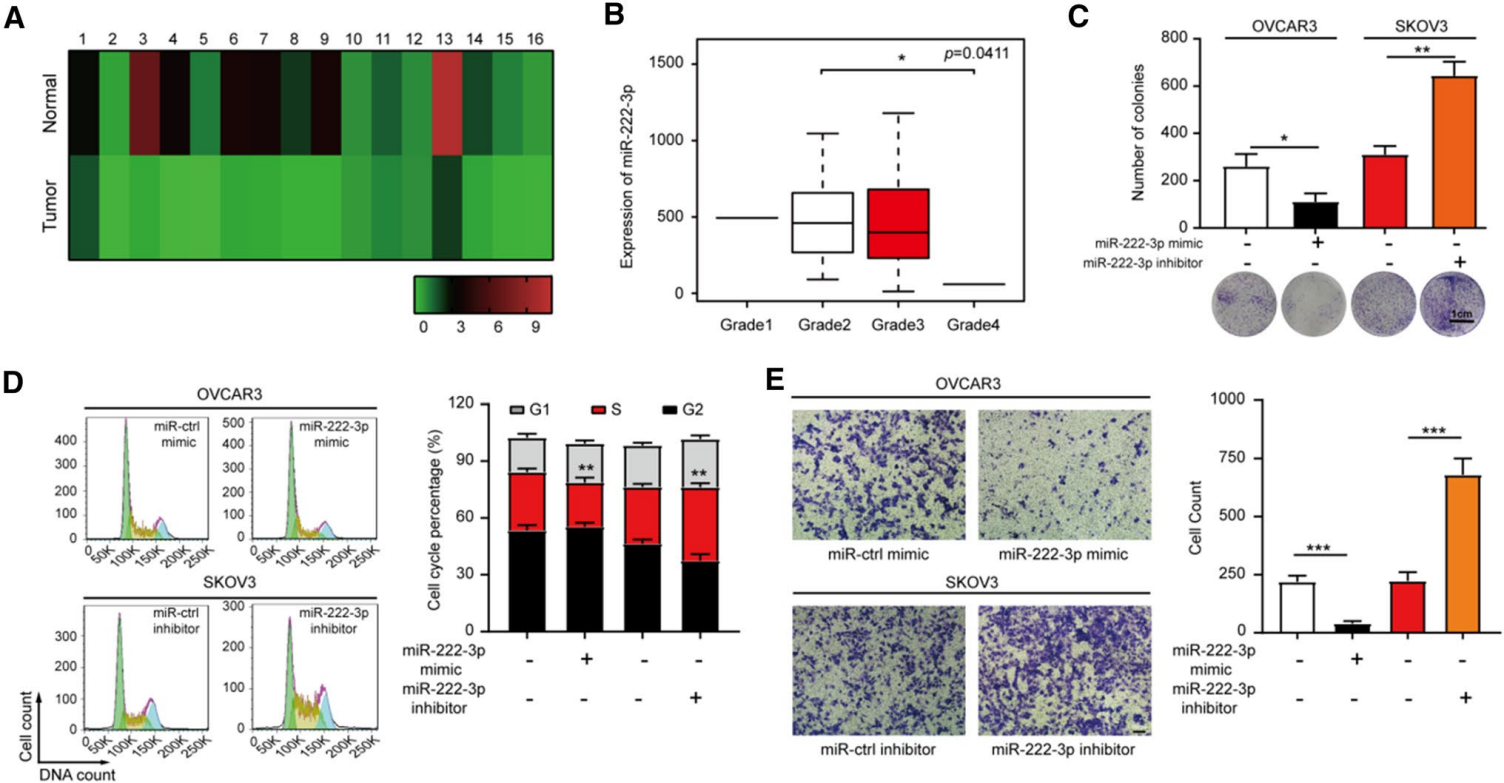
miR-222-3p was significantly down-regulated in OC and inhibited cell proliferation and migration in vitro. **A** miR-222-3p was significantly down-regulated in OC tissues (*p* < 0.0001). **B** The correlation between miR-222-3p expression level and each grade in OC was analyzed by TCGA-miRNA-FPKM transcriptomic data (*n* = 379). **C, D** Colony formation assay (**C**) and flow cytometry assays (**D**) revealed that miR-222-3p could affect the proliferation of OC (OVCAR3, SKOV3) cells. (**E**) Transwell assay revealed that miR-222-3p could affect the migration of OC (OVCAR3, SKOV3) cells. All data (**C–E**) represent the mean ± SD in different assays, revealed by unpaired two-tailed t-test

Our lab has previously reported that miR-222-3p could target GNAI2 to inhibit tumor proliferation and target PDCD10 to inhibit cell migration in OC [6, 7]. However, the underlying upstream mechanisms of miR-222-3p remain unclear. First, we detected the expression level of miR-222-3p in normal ovarian tissues and tumor tissues and found that the expression of miR-222-3p in OC tissues was significantly down-regulated (*p* < 0.0001; Fig. 1A). In addition, according to the expression of miR-222-3p in the TCGA-miRNA-FPKM transcriptomic data (379 OC samples), the grade of OC was divided into grades 1–4, and it was found that the expression level of miR-222-3p was correlated with the grade of OC patients. That is, the expression level of miR-222-3p was down-regulated with grade in 379 OC samples, and the difference in expression of miR-222-3p in stage 2 and stage 4 was the most obvious (*p* = 0.0411; Fig. 1B).

In this study, we also conducted a series of in vitro experiments to clarify the role of miR-222-3p in inhibiting the migration and proliferation of cancer cells. We first detected the expression levels of miR-222-3p in four OC cell lines (OVCAR3, SKOV3, HO-8910 PM, and HO-8910**) **(Fig. S1A). Subsequently, miR-222-3p was then overexpressed in OVCAR3 and HO-8910 PM cells and down-regulated in SKOV3 and HO-8910 cells. It was found that the proliferation ability of miR-222-3p mimic group was lower than that of miR-ctrl mimic group in colony formation assay (Fig. 1C) and flow cytometry assays (Fig. 1D). On the contrary, the miR-222-3p inhibitor group showed a higher proliferation ability compared with that in the miR-ctrl inhibitor group. Similarly, miR-222-3p was overexpressed in HO-8910 PM cells and down-regulated in HO-8910 cells also obtained the results described above (Fig. S1B and S1C). Moreover, the miR-222-3p mimic inhibited OC cells (OVCAR3 and HO-8910 PM) migration in the Transwell assay. In contrast, the miR-222-3p inhibitor group had a higher migration ability to OC cells (SKOV3 and HO-8910) than the miR-ctrl inhibitor group (Fig. 1E and S1D). These experimental results showed that miR-222-3p was low expressed in OC and could suppress the proliferation and migration of OC cells.

### miR-222-3p directly suppresses CDK19 expression by binding to its 3′-UTR and inhibits OC cell proliferation

**Fig. 2:**
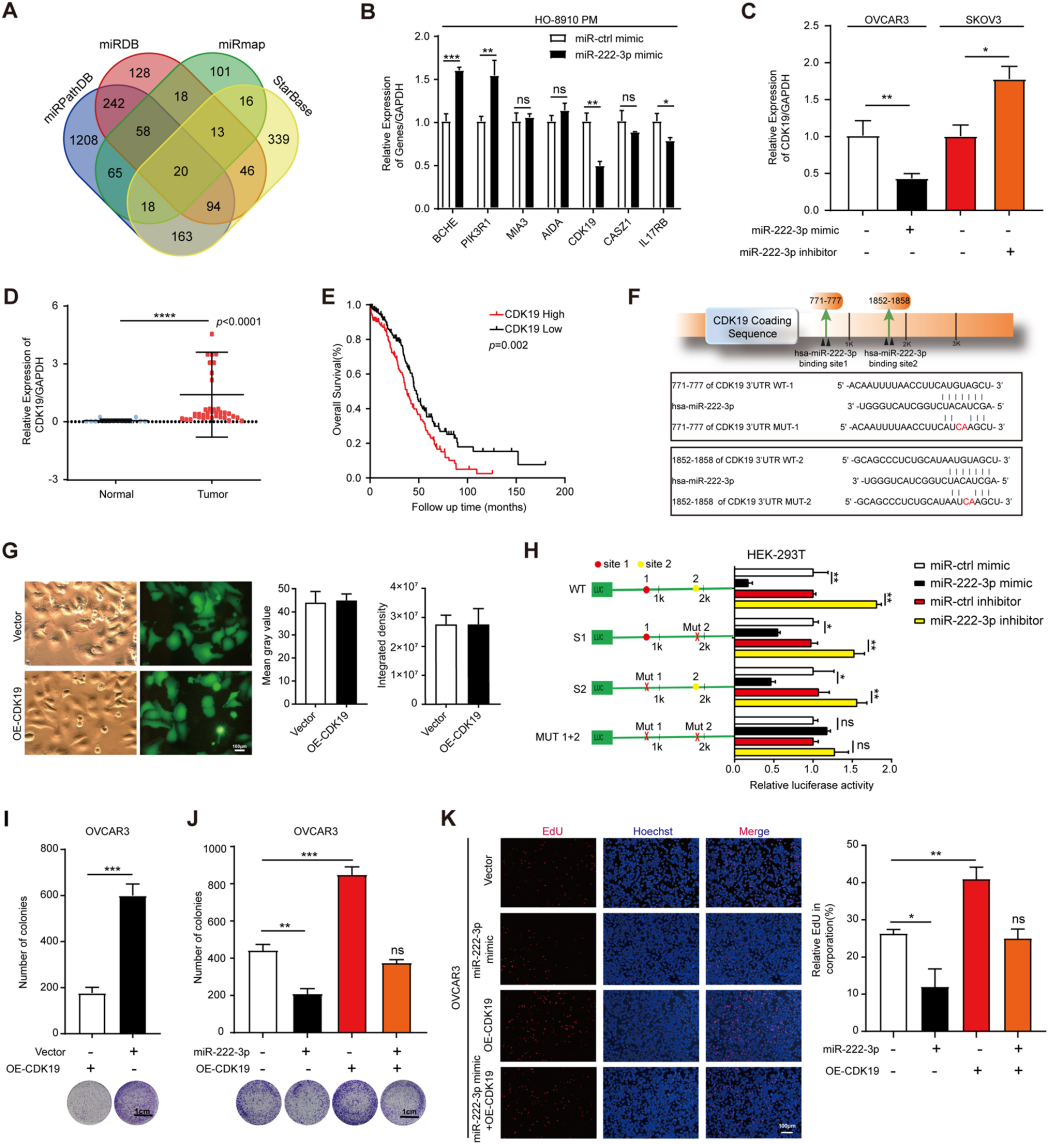
miR-222-3p directly suppresses CDK19 expression by binding to its 3’-UTR and inhibits OC cell proliferation.** A** A Venn diagram was intersected to look for the downstream candidate genes targeted by miR-222-3p. **B** Expression levels of seven candidate genes were detected after transfection with miR-222-3p mimic in OC cells. **C** qRT-PCR analyses were used to detect CDK19 mRNA levels after transfection with miR-222-3p mimic or inhibitor in OVCAR3 cells or SKOV3 cells, respectively, the results were determined by an unpaired two-tailed *t*-test. **D** CDK19 was significantly up-regulated in OC tissues (*p* < 0.0001). **E** Kaplan–Meier curves for overall survival probability in OC patients with low and high CDK19 expression. **F** Schematic description of the imaginary double strand formed by miR-222-3p with the 3′-UTR of CDK19. **G** Effectiveness of the CDK19 and ctrl vector with GFP fluorescence (*left*), the quantized figure is the Mean gray value (*middle*) and Integrated density (*right*). **H** Relative luciferase activities in HEK-293T cells co-transfected with a miR-222-3p mimic/or inhibitor and CDK19 WT/or MUT. **I** The proliferation ability of OVCAR3 after CDK19 transfection was assessed by the colony formation assay, the results were determined by an unpaired two-tailed *t*-test. **J, K** The colony formation assay (**J**) and EdU assay (**K**) revealed inhibition of proliferation when OVCAR3 cells were transfected with miR-222-3p mimic. Recovery assays showed that miR-222-3p suppressed the proliferation of OVCAR3 cells due to its inhibitory effect on CDK19. The results of J and K are presented as mean ± SD and determined by unpaired two-way ANOVA. All the experiments were performed in triplicate

We used the online databases miRDB, miRPathDB, miRmap, and StarBase to predict target candidate genes downstream of miR-222-3p. As shown in Fig. 2A, we obtained 20 candidate genes through intersection screening (Fig. 2A). We selected several molecules strongly correlated with miR-222-3p and literature closely related to proliferation or migration through the online database, and a total of 7 genes were selected. Next, we transfected miR-222-3p mimic into the cells, and qPCR assay was used to detect the expression levels of seven candidate genes. As shown in Fig. 2B, compared with the miR-ctrl mimic group, two genes were significantly up-regulated and down-regulated in each group transfected with miR-222-3p mimic, while the expression of three genes had no significant difference. We focused on the gene down-regulated genes, among which the expression level of CDK19 was down-regulated more significantly (Fig. 2B). Therefore, in our research, CDK19 was selected for further analysis.

Cyclin-dependent kinase 19 (CDK19) is an important regulator in the process of cancer, and CDK19 inhibitors can be used as a kinase inhibitor for cancer treatment [9]. Then we have examined both CDK19 mRNA and protein levels in IOSE and four different OC cell lines and found that CDK19 mRNA and protein levels were highly expressed in the OC cells (Fig. S2A). These results suggested that CDK19 played an oncogenic role in OC. Moreover, we used qRT-PCR to detect the expression of CDK19 mRNA, and it was found that the CDK19 mRNA level was decreased in the group transfected with miR-222-3p mimic in OVCAR3 cells, while the CDK19 mRNA level was increased in the group transfected with miR-222-3p inhibitor in SKOV3 cells (Fig. 2C). We also found CDK19 was down-regulated at the protein level by miR-222-3p mimic, whereas miR-222-3p inhibitor elevated the CDK19 protein expression (Fig. S2B). These results indicated that miR-222-3p has an inhibitory effect on CDK19 expression.

Besides, CDK19 was highly expressed in OC tissues (Fig. 2D), and we analyzed the correlation between CDK19 expression level and survival time of OC patients in the TCGA database, and the results showed that CDK19 high expression was often associated with poor prognosis of OC patients (Fig. 2E). Moreover, the meta-analysis depicting forest plots indicated that CDK19 expression levels were associated with poor patient outcomes in the GEO datasets (Fig. S2C). In addition, we used CDK19 antibody to perform IHC staining on randomly selected OC tissues and normal ovarian tissues and found that compared with normal ovarian tissues, CDK19 was expressed higher in OC tissues (Fig. S2D).

CDK19 was predicted to have two miR-222-3p binding sites in its 771–777 and 1852–1858 3′-untranslated region (3′-UTR) by Targetscan (Fig. 2F). We used the dual-luciferase assay to determine the binding site of the predicted seed sequence. First, we verified the transfection efficiency of CDK19 by GFP fluorescence detection (Fig. 2G). Then, we constructed the luciferase reporter gene containing either wild-type CDK19 771–777/or 1852–1858 3′-UTR (CDK19 3′-UTR WT-1/or -2) binding site or mutated CDK19 771–777/or 1852–1858 3′-UTR (CDK19 3′-UTR MUT-1/or -2) binding site for miR-222-3p, respectively (Fig. 2H). Luciferase reporter assay revealed that the luciferase activity of CDK19 3′-UTR WT-1/or -2 was significantly reduced after increasing the levels of miR-222-3p by treating HEK-293T cells with miR-222-3p mimic, while the luciferase activity of CDK19 3′-UTR WT-1/or -2 was significantly increased after reducing the levels of miR-222-3p by treating HEK-293 T cells with miR-222-3p inhibitor (Fig. 2H). However, CDK19 3′-UTR MUT1/or -2 was co-transfected into HEK-293T cells with miR-222-3p mimic or miR-222-3p inhibitor, and the luciferase activity was not affected by miR-222-3p only when both binding sequences were mutated (Fig. 2H). These results suggested that miR-222-3p could inhibit CDK19 expression by directly binding to its 3’-UTR.

Next, we investigated the function of CDK19. Both colony formation assay (Fig. 2I) and EdU assay **(Fig. S2E)** showed that CDK19 overexpression could promote the proliferation of OVCAR3 cells. To determine whether miR-222-3p inhibits the proliferation of OC cells due to its targeting of CDK19, we designed the following recovery experiment. In colony formation assay and EdU assay, the overexpression of CDK19 eliminated the inhibitory effect of miR-222-3p mimic on OVCAR3 cell proliferation (Fig. 2J, K). As expected, colony formation assays and flow cytometry assays showed that overexpression of CDK19 could also eliminate the inhibitory effect of miR-222-3p mimic on HO-8910 PM cell proliferation (Fig. S2F and S2G). In addition, CDK19 mRNA and protein levels were higher in the group co-transfected with miR-ctrl mimic and CDK19 than in the other groups. Similarly, overexpression of CDK19 in HO-8910 PM cells effectively restored the CDK19 mRNA and protein levels suppressed by miR-222-3p mimic (Fig. S2H).

### miR-222-3p suppresses OC tumor growth in vivo by targeting CDK19

**Fig. 3:**
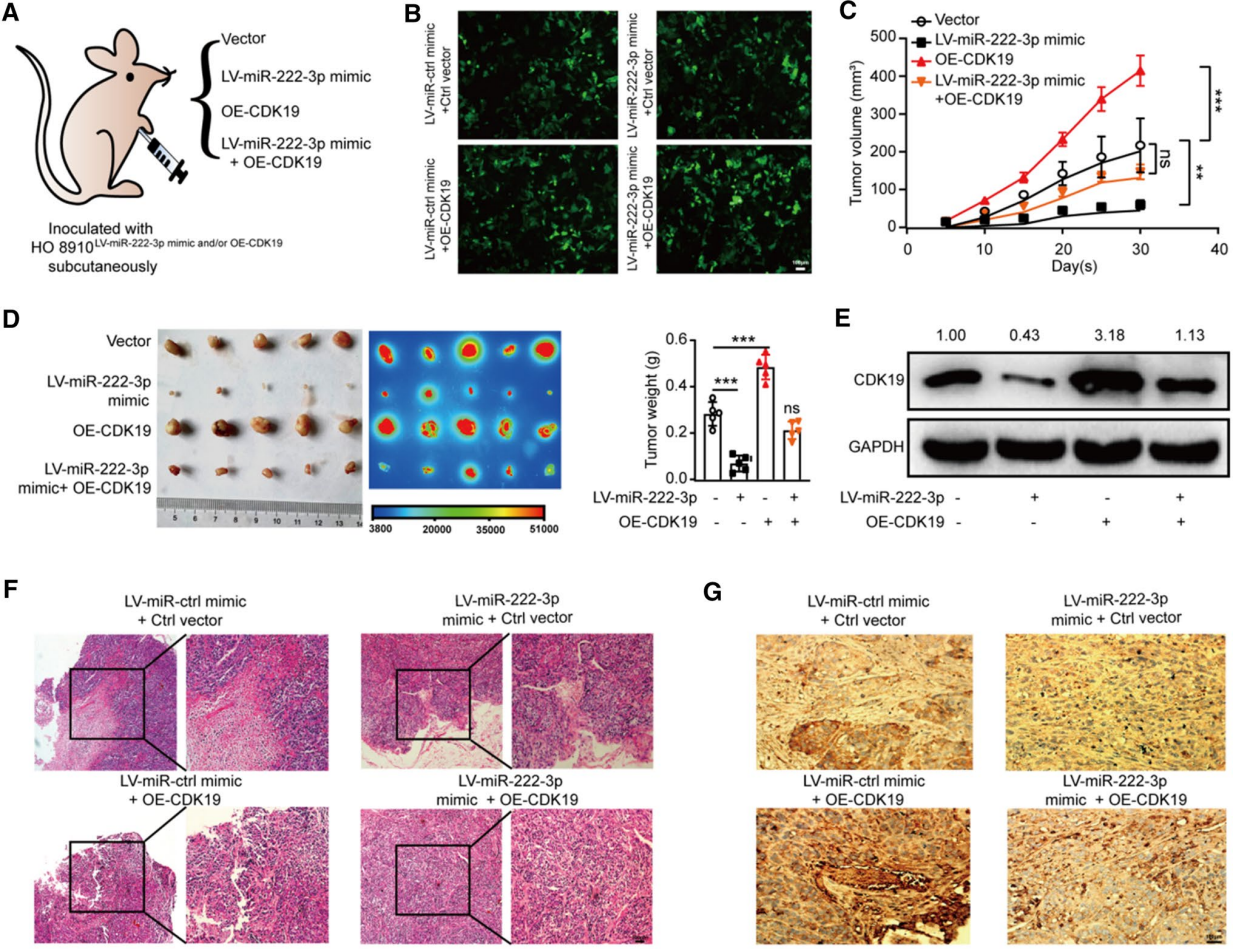
miR-222-3p suppresses OC tumor growth in vivo by targeting CDK19. **A** Subcutaneous xenograft model. The equivalent numbers of LV-miR-222-3p and GFP-labeled OE-CDK19 transfected stably in OC cells (1 × 10^7^) were subcutaneously (s.c.) injected into each mouse (*n* = 5). Tumor growth was monitored every 5 days. **B** Fluorescence efficiency of four groups of stable cell lines. **C** The volume curve of tumor growth in mice for 30 days. **D** Representative images and bioluminescence images of tumors in mice 30 days after implantation (*n* = 5 mice per group, *right*). *Bar*, 0.5 cm. The tumor weights in nude mice at the 30 days were determined (*left*). **E** Western blotting analysis of CDK19 levels in OC xenografted tumor. Image J calculated the relative expression rate. **F** Representative HE staining of the tumor tissues was obtained from 30 days after implantation. Bar, 50 µm (*left*) and 100 µm (*right*). **G** IHC staining for CDK19 in the tumor tissues of mice 30 days after implantation. *Bar*, 100 µm. Results are presented as mean ± SD. The results of (**C, D**) are presented as mean ± SD and determined by unpaired two-way ANOVA

To verify the relationship between miR-222-3p and CDK19 in vivo, we subcutaneously injected with lentivirus-miR-222-3p (LV-miR-222-3p) and GFP-labeled OE-CDK19 luciferase-expressing stable cells into each nude mice (*n* = 5) (Fig. 3A, B). The group transfected with LV-miR-222-3p showed a significant inhibitory effect on tumor growth than the OE-CDK19 and LV-miR-222-3p co-transfected group. Restoration of CDK19 expression reversed the inhibition of tumor growth by miR-222-3p (Fig. 3C, D). Similarly, Western blotting analysis of CDK19 proteins in the tumors showed that the OE-CDK19 effectively restored CDK19 protein levels inhibited by miR-222-3p (Fig. 3E). Moreover, H&E staining revealed lower stroma-rich architecture in tumor tissues in the LV-miR-222-3p and CDK19 co-transfected group compared with the LV-miR-ctrl OE-CDK19 co-transfected group (Fig. 3F). The IHC staining of the tumor of mice showed that the expression of CDK19 protein was significantly increased in the LV-miR-ctrl and OE-CDK19 co-transfected group, while the expression of CDK19 protein was reversed in the LV-miR-222-3p and CDK19 co-transfected group (Fig. 3G). Thus, our in vivo experimental data showed that the miR-222-3p/CDK19 regulatory axis was negatively correlated with OC growth.

### Hotair can sponge miR-222-3p and has a negative correlation with miR-222-3p

**Fig. 4:**
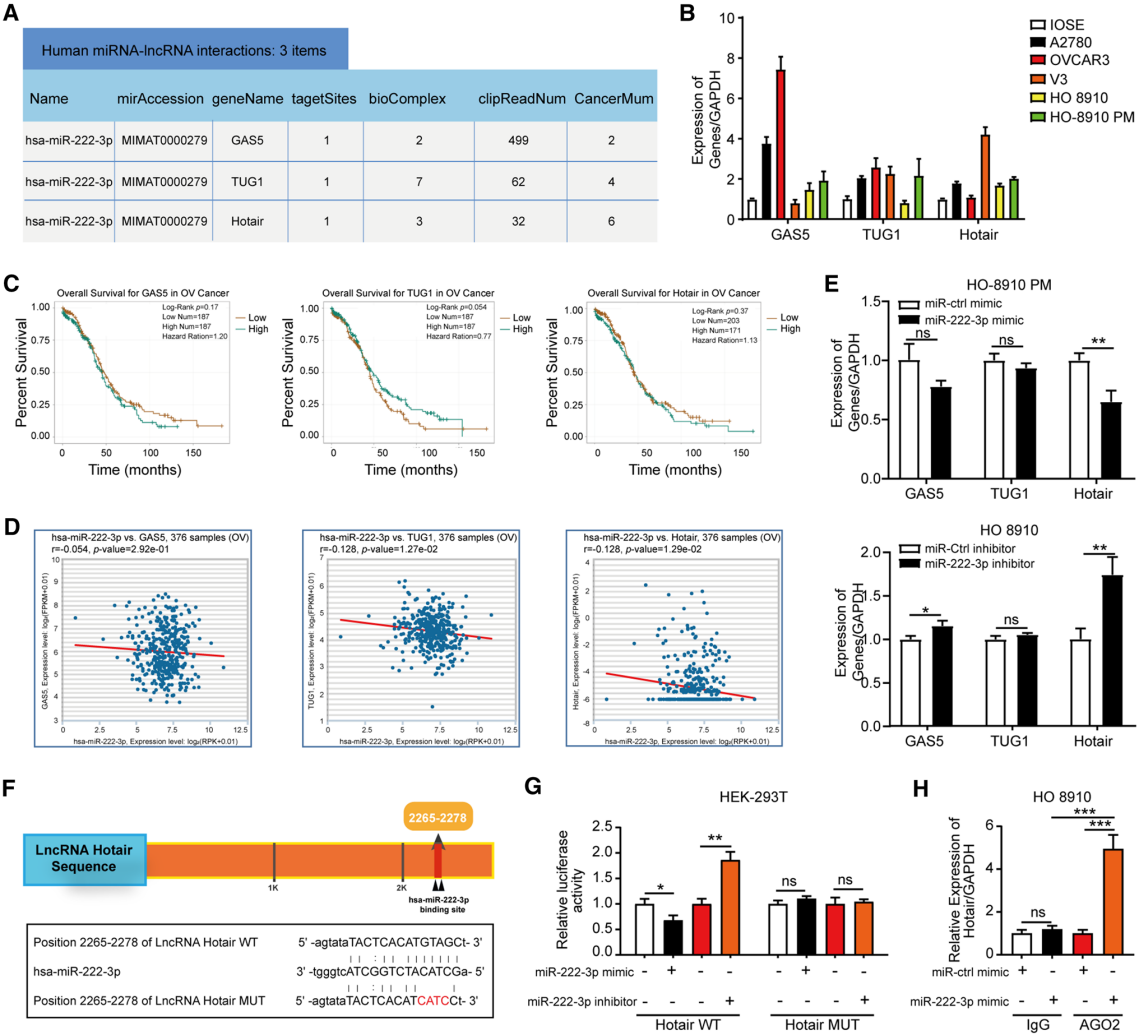
Hotair can sponge miR-222-3p and has a negative correlation with miR-222-3p. **A** The LncRNAs targeting miR-222-3p predicted by StarBase v2.0 (Screening Condition: Clade: mammal; Genome: Human; Assembly: hg19; Number of Cancer Types > 2). **B** qPCR analyses of GAS5, TUG1, and Hotair levels in IOSE and five different OC cell lines. **C, D** The survival prognosis of GAS5, TUG1, and Hotair in OC and its correlation with miR-222-3p were analyzed by StarBase 3.0. **E** HO-8910 PM and HO-8910 cells were transfected with miR-222-3p mimic and miR-222-3p inhibitor, respectively, and the expression levels of GAS5, TUG1, and Hotair were detected, the results were determined by an unpaired two-tailed *t*-test. **F** Schematic of predictive binding sites for Hotair, and the site mutation design for the reporter gene assay. **G** The relative luciferase activities in the HEK-293T cells co-transfected with miR-222-3p mimic/or inhibitor, the reporter vector Hotair wild-type (Hotair-WT), or the reporter vector Hotair mutation (Hotair-MUT). **H** RIP assays were performed using an anti-AGO2 antibody with the transfection of miR-222-3p mimic or miR-ctrl mimic in HO-8910 cells to detect Hotair expression according to qRT-PCR. The results are presented as mean ± SD and determined by unpaired two-way ANOVA

Many studies have reported that many LncRNAs could function as competing endogenous RNAs (ceRNAs) to regulate gene expression by competing for miRNAs binding [21, 22]. As per the StarBase v2.0 database, three LncRNAs targeting miR-222-3p were found (Fig. 4A). We have examined the mRNA levels of GAS5, TUG1, and Hotair in IOSE and five different OC cell lines (Fig. 4B). We found that mRNA levels of these three LncRNAs in normal ovarian cells were lower than those in the OC cells. The results indicated that these three LncRNAs had an oncogenic role in OC. Next, we analyzed the prognostic survival of these three LncRNAs in OC and their correlation with miR-222-3p through the online database. It can be seen that the HR of GAS5 (HR = 1.20) and Hotair (HR = 1.13) is greater than 1, indicating that they can be used as independent prognostic genes in OC (Fig. 4C). In addition, the correlation analysis figures with miR-222-3p showed that Hotair had a higher correlation with miR-222-3p (Fig. 4D). We also investigated the correlation between these three lncRNAs and miR-222-3p by qRT-PCR assay and found that after transfection of miR-222-3p mimic in the HO-8910 PM, only the expression level of Hotair decreased significantly (Fig. 4E), while after transfection of miR-222-3p inhibitor, both GAS5 and Hotair expression levels increased, but Hotair changed most significantly. Therefore, in the study, Hotair was selected as the upstream of miR-222-3p for further exploration in combination with the results of bioinformatics analysis and qRT-PCR assay.

StarBase v2.0 predicted that miR-222-3p harbors the complementary binding sequence of Hotair (Fig. 4F). Based on the sequence, we constructed the reporter vector Hotair wild-type (Hotair-WT) plasmid and Hotair-binding site mutation (Hotair-MUT) plasmid for the dual-luciferase reporter assays (Fig. 4G). It was further revealed that the overexpression of miR-222-3p significantly reduced luciferase activity in the Hotair-WT group, while inhibition of miR-222-3p promoted luciferase activity. When the luciferase assay was repeated with the Hotair-MUT plasmid, miR-222-3p mimic or inhibitor did not affect luciferase activity (Fig. 4G). It indicates that miR-222-3p and Hotair can combine. Moreover, RNA immunoprecipitation (RIP) experiment was used to verify whether miR-222-3p was directly associated with Hotair. We conducted anti-AGO2 RIP in HO-8910 cells transiently up-regulating miR-222-3p. Hotair pull-down by AGO2 was specifically enriched in miR-222-3p treated cells, validating that miR-222-3p was a bona fide Hotair targeted miRNA (Fig. 4H). Similarly, the AGO2 immunoprecipitation assay showed that the presence and significant enrichment of Hotair and miR-222-3p in AGO2 pull-down products compared with the negative control. This indicates that AGO2 can be recruited by Hotair, and indicating that miR-222-3p can pull down Hotair in an AGO2-dependent manner, further verifying their binding potential (Fig. S3A). These results indicate that Hotair can sponge miR-222-3p and has a negative correlation with miR-222-3p.

### Hotair can promote the proliferation and migration of OC in vitro, and promote the growth and migration of OC in vivo

**Fig. 5:**
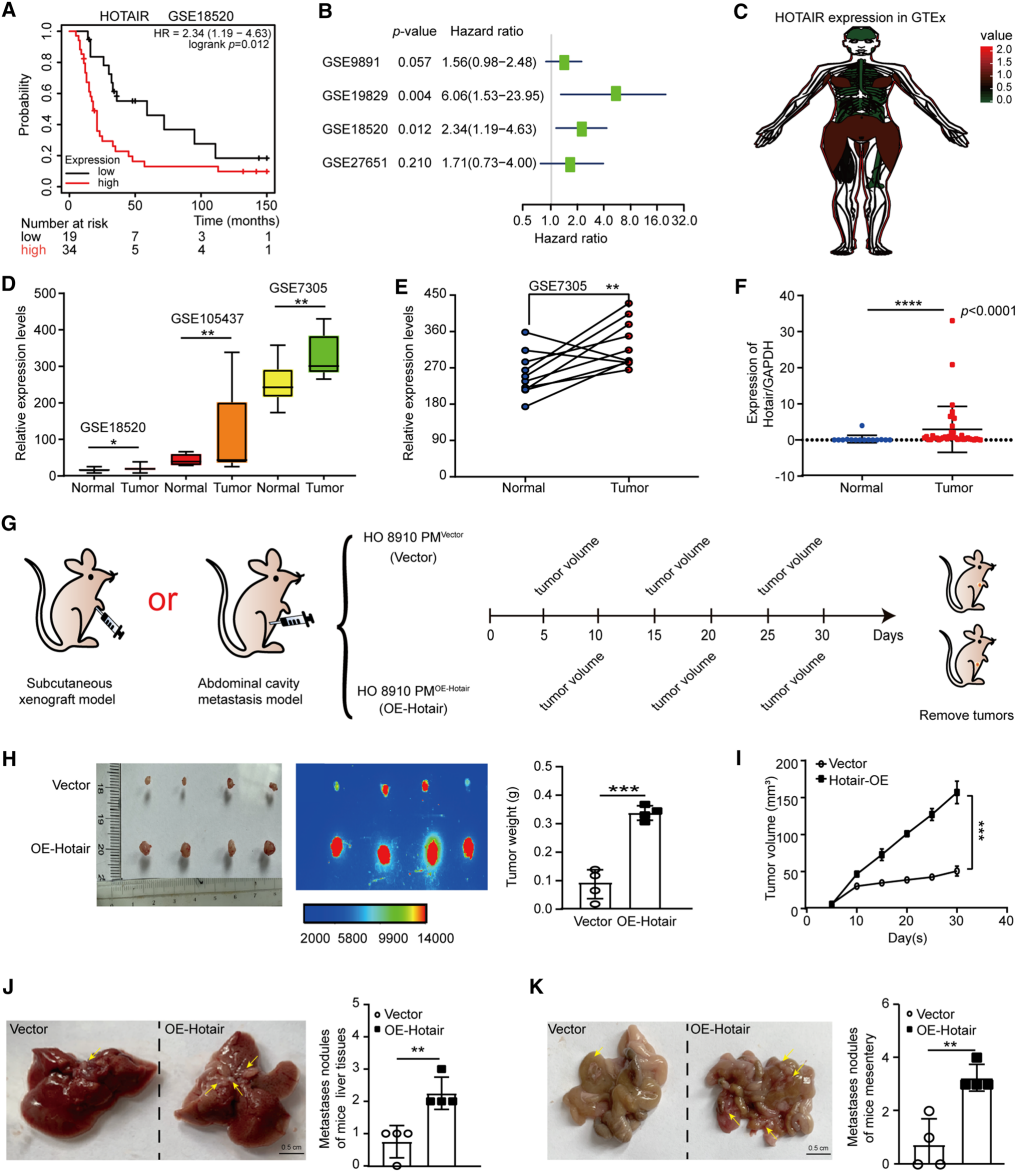
Hotair can promote the proliferation and migration of OC in vitro, and promote the growth and migration of OC in vivo.** A** Kaplan–Meier survival curves were produced for high (*red line*) and low (*blue line*) Hotair expression in the OC samples in the GSE 18,520 dataset. Hotair expression was associated with poor overall survival (OS) in OC (*p* = 0.012). **B** Meta-analysis depicting forest plots of Hotair expression as a univariate predictor of overall survival. **C** Hotair expression in female body organs of normal tissue samples in GTEx database. **D** The expression of Hotair in OC samples and normal ovary samples from GSE18520, GSE105437, and GSE7305 datasets. N stands for normal, C stands for cancer. **E** Hotair expression in ovarian cancer and paired normal samples from the GSE7305 dataset. **F** qRT-PCR was used to detect Hotair expression in normal ovary tissue and OC tissue. **G** The abdominal cavity metastasis model and subcutaneous xenograft model. HO-8910 PM^Vector/or OE−Hotair^ stable cells (1 × 10^7^) were subcutaneously (s.c.) and intraperitoneally (i.p.) injected into each mouse. **H** The representative images and bioluminescence images from nude mice inoculated in Vector and OE-Hotair (*n* = 4 mice per group, *left*). *Bar*, 0.5 cm. The tumor weights in nude mice at the 30 days were determined (*right*). **I** The tumor volumes were measured every 5 days after injection. **J, K** Representative images (*left*) and metastatic nodule plots (*right*) of mice liver tissues **(J)** and mesentery **(K)**. Bar, 0.5 cm. Results are presented as mean ± SD. *p < 0.05, **p < 0.01, ***p < 0.001, ****p < 0.0001. The results (**H–K**) were determined by an unpaired two-tailed *t*-test

Hotair was up-regulated in OC and played an important role in tumor development [19, 23]. We analyzed the correlation between Hotair expression and survival times in OC patients from GSE18520, using the Kaplan–Meier plotter public database, and the results showed that high expression of Hotair was often associated with poor prognosis in OC patients (Fig. 5A). Furthermore, meta-analysis depicting forest plots indicated that Hotair was a high-risk molecule in the GEO datasets of OC patients (Fig. 5B). The analysis of Hotair expression in female body organs of normal tissue samples in the GTEx database, Hotair is relatively high in the ovary (Fig. 5C). In addition, analysis of Hotair expression in three GEO datasets (GSE18520, GSE105437, GSE7305), Hotair significantly up-regulated expression in OC samples (Fig. 5D, E). Besides, significant up-regulation of Hotair expression was detected when OC tissue samples, were compared with healthy tissues (Fig. 5F). These results suggest that Hotair is a potential oncogene in OC.

Then, we investigated the regulation of Hotair on OC at the cellular level. We used overexpression (OE) plasmid to specifically up-regulate Hotair expression in OC cells. From flow cytometry assays (Fig. S3B and S3D), colony formation assay (Fig. S3C), EdU assay (Fig. S3E), and Transwell assays (Fig. S3F and S3G), we can conclude that Hotair can promote the proliferation and migration of OVCAR3 and HO-8910 PM cells. We next constructed the abdominal cavity metastasis model and subcutaneous xenograft model to explore the effects of Hotair on tumor growth and metastasis (Fig. 5G). The result of subcutaneous injection was shown in Fig. 5H, I, and the result of intraperitoneal injection was shown in Fig. 5J, K. Hotair overexpression (OE-Hotair) significantly increased the mean tumor weight (Fig. 5H) and average tumor volume (Fig. 5I) as compared with the Vector group. To assess the effects of Hotair on tumor cell colonization and dissemination, the abdominal cavity metastasis models were constructed. We injected mice with stable cells (*n* = 4) into the abdominal cavity. Meanwhile, in the abdominal cavity metastasis model, we observed that OE-Hotair dramatically increased the number of metastatic nodules in liver and mesentery tissues (Fig. 5J, K). These data revealed that Hotair promoted tumor growth and metastasis*.*

### Hotair regulates CDK19 expression via miR-222-3p

**Fig. 6:**
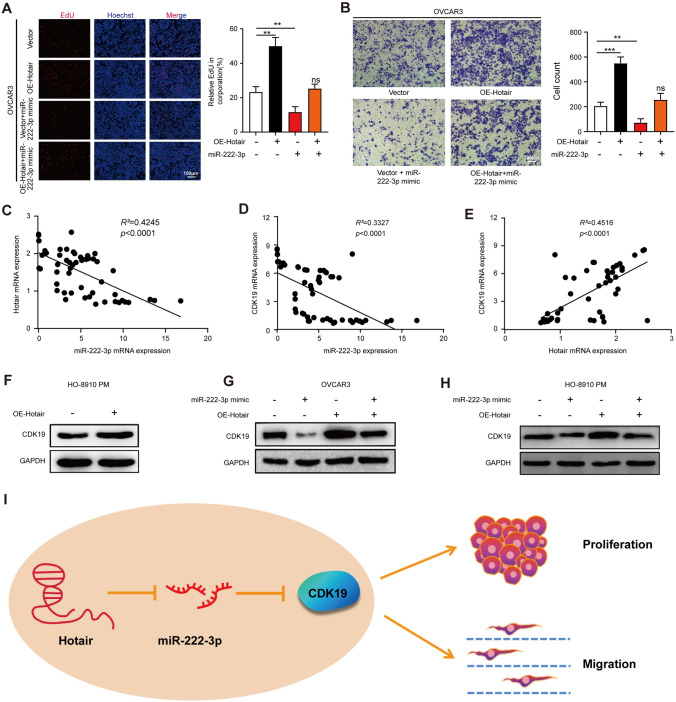
Hotair regulates CDK19 expression via miR-222-3p. **A** EdU assay was used to detect the rescue effect of OE-Hotair on OVCAR3 cells proliferation abilities (*left*). *Scale bar*, 100 µm. The number of cells was counted (*right*). **B** Transwell assay was used to detect the rescue effect of OE-Hotair on OVCAR3 cells’ migration abilities. *Scale bar*, 100 µm (*left*). The number of cells was counted (*right*). **C–E** Pearson’s correlation scatter plots show the fold changes of CDK19 mRNA, miR-222-3p miRNA, and Hotair mRNA levels in OC tissues (*n* = 54). **F** After the transfection of the OE-Hotair plasmid, the protein expression of CDK19 was detected by Western blotting. **G, H** Western blotting of CDK19 in OVCAR3 and HO-8910 PM cells with Hotair and/or miR-222-3p mimics. **I** A working model describing the interaction between Hotair/miR-222-3p/CDK19 during cancer development. Hotair regulated CDK19 by sponging miR-222-3p expression and subsequent targeting of genes. Results are presented as mean ± SD

Previously, we have confirmed that Hotair can bind to miR-222-3p and the expression level is negatively correlated. To explore the functional correlation between Hotair and miR-222-3p, we used EdU assay (Fig. 6A) and Transwell assay (Fig. 6B) to perform a functional recovery experiment. We found the promoted cell migration and proliferation in OE-Hotair transfected OVCAR3 cells were attenuated by co-transfection with miR-222-3p mimic. The same results were confirmed in HO-8910 PM cells by the flow cytometry assay (Fig. S3H), EdU assay (Fig. S3I), and Transwell assay (Fig. S3J). Overall, these results suggested that Hotair promotes cell proliferation and migration in OC cells by sponging miR-222-3p.

Many studies have reported that LncRNAs could function as ceRNAs to regulate target gene expression by competing for miRNAs binding [21, 22]. Thus, we sought to investigate whether Hotair could regulate CDK19 levels by competitively binding to miR-222-3p in OC cells. We performed the qRT-PCR analysis of Hotair, miR-222-3p, and CDK19 in 54 OC patients and observed a strong correlation between Hotair and CDK19 expression levels **(**Fig. 6C–E). In vitro experiments, Western blotting results showed that CDK19 expression was up-regulated in the OE-Hotair group in OC cells (Fig. 6F). As shown in Fig. 6G, H, miR-222-3p mimic reduced the expression of CDK19, whereas Hotair substantially eliminated this effect in OVCAR3 and HO-8910 PM cells (Fig. 6H). In summary, our data indicated that Hotair could regulate CDK19 levels by competitively binding to miR-222-3p in OC cells.

Our findings are depicted as a cartoon presented in Fig. 6I. Our study has unveiled Hotair sponging miR-222-3p expression and indirectly up-regulates CDK19 expression thus promoting the occurrence and development of OC.

## Discussion

OC’s mortality rate is the first among gynecological tumors and the clinical efficacy is not ideal [24]. It is important to fully understand the molecular mechanisms involved in OC development and identify novel prognostic predictors. In our study, we provided evidence from a large of OC data sets along with in vitro and in vivo experiments. We first found that miR-222-3p is a tumor-suppressor molecule in OC and could promote the migration and proliferation of OC cells. Then our data showed that miR-222 could directly repress CDK19 expression by binding to its 3’UTR. In addition, this study showed Hotair also promoted the growth of tumors in vivo. We have also identified Hotair via miR-222-3p/CDK19 axis regulate OC development, which has important implications for our understanding of OC growth and metastasis. Altogether, we demonstrated that Hotair promotes the biological malignancy of OC by sponging miR-222-3p and regulating CDK19 expression (Fig. 6I).

MicroRNAs (miRNAs) are a class of endogenous single-stranded small RNAs and do not encode proteins. They are coded by genome and are about 17–25 nucleotides in length [4]. They are widely involved in regulating cell proliferation, differentiation, apoptosis, growth, and development [25]. miRNAs are abnormally expressed in many cancer types, including prostate cancer [26], colorectal cancer [27], colon cancer [28], breast cancer [29], liver cancer [30], gastric cancer [31], etc. There is also a close link between miRNAs and OC tumorigenic processes [5]. Mature miR-222 sequences have a hairpin precursor with different arms called the 5′ or 3′ arm, which are also known as -5p or -3p, respectively. miR-222-3p is processed from the arm of the 3′ end of the precursor [32, 33]. Our laboratory has previously reported that miR-222-3p overexpression could inhibit the proliferation and migration of OC cells and is positively correlated with a good prognosis of OC [7]. However, the functions of miR-222-3p in OC still need to be further elucidated.

Cancer is a pathological manifestation of uncontrolled cell division; therefore, researchers have long believed that understanding the basic principles of cell cycle control will contribute to the treatment of cancer. Cyclin-dependent kinases (CDKs) are critical regulatory enzymes that drive all cell cycle transitions [34], and their activity is under stringent control to ensure successful cell division. In particular, CDK promotes cell cycle transition and is considered as a key therapeutic target for cancer [35]. Selective inhibition of CDK may provide a new treatment for some cancers [9], so CDK inhibitors have become tumor therapeutics. Studies have shown that CDK8/19 inhibits premature G1/S conversion and ATR-dependent cell death in inducible prostate cancer cells [11]. Studies have also shown that the mediator kinase CDK8/CDK19 drives Yap1-dependent BMP4-induced EMT in tumors [12]. The role of CDK19 in tumorigenesis is rare, and its role in ovarian cancer has not been reported. Therefore, studying the role of CDK19 in OC will help us to further understand the molecular mechanism of ovarian cancer development and provide new ideas for judging prognosis and treatment.

Herein, we found that miR-222-3p acted as a tumor-suppressor to inhibit OC cell migration and proliferation in vitro, and inhibit tumor metastasis and growth by targeting CDK19, revealing the significance of miR-222-3p and CDK19 in OC. It would be worth exploring the miR-222-3p/CDK19 regulatory axis as a treatment strategy for OC.

At present, the LncRNA-miRNA-mRNA regulatory network is widely recognized. In this network, LncRNA can sponge miRNA, thereby regulating the expression of miRNA targets genes and affecting post-transcriptional regulation [36, 37]. LncRNA is a class of RNA molecules with transcript length exceeding 200 nt and does not encode proteins [13, 38]. Some studies have shown that Hotair was expressed unusually in many cancers, including OC, and it is closely related to the treatment of OC [39]. We also found that high Hotair expression was associated with OC proliferation and metastasis [40].

In our study, we discovered that Hotair shared a unique sequence complementary to miR-222-3p through bioinformatics analysis, which was further validated by luciferase reporter assay and RIP assay. In addition, the expression of Hotair was negatively associated with miR-222-3p in OC. Then, we conducted a series of functional experiments and found that Hotair can regulate the biological function of OC in vivo and in vitro. We also predicted miR-222-3p targeted CDK19 through the online database. Moreover, OE-Hotair can lead to increased expression of CDK19. Hence, we further provided evidence for the regulation of CDK19 by Hotair in OC. These results showed that Hotair positively regulated the expression of CDK19 through sponging miR-222-3p, which enhances the malignant behavior in OC. Many studies to date have been reported on the role of miR-222-3p paralogue miR-221-3p in cancer development either as oncomiR or as oncosuppressor-miRs, and also plays a tumor-suppressive role in OC [41, 42]. Similarly, CDK8 has also been reported to promote carcinogenesis in OC [43]. We found through the online database that Hotair has the binding motif with miR-221-3p, and miR-222-3p also has the binding seed sites with 3’UTR of CDK8. Our results could only show that Hotair can sponge miR-222-3p to regulate CDK19 in OC, however, we do not know whether miR-221-3p and CDK8 have some effect in this regulatory axis or not.

Altogether, we find that Hotair plays an oncogenic role in OC development via the miR-222-3p/CDK19 axis. The Hotair/miR-222-3p/CDK19 axis plays an important role in OC and drives the malignant transformation of OC cells. The results of this study may provide attractive potential targets for the prevention and treatment of OC.

## Supplementary Information

Below is the link to the electronic supplementary material.

Supplementary file1 (DOC 13402 KB)

## Data Availability

All data generated or analyzed during this study are included in this published article and its supplementary information files.

## Abbreviations

LncRNAs: Long non-coding RNAs
Hotair: HOX transcript antisense RNA
OC: Ovarian cancer
3′-UTR: 3′-Untranslated region
qRT-PCR: Quantitative real-time polymerase chain reaction
CDK: Cyclin-dependent kinase
NTA: Nanoparticle-tracking analysis
PBS: Phosphate-buffered saline
TCGA: The cancer genome atlas
GSEA: Gene set enrichment analysis
HEK: Human embryonic kidney
GO: Gene ontology
KEGG: Kyoto encyclopedia of genes and genomes
DMEM: Dulbecco’s modified eagle’s medium
SDS-PAGE: Sodium dodecyl sulfate–polyacrylamide gel electrophoresis
FBS: Fetal bovine serum
RIP: RNA immunoprecipitation
AGO2: Argonaute2
IgG: Immunoglobulin G
PI: Propidium iodide
ceRNA: Competing endogenous RNA
