# Supplementary material for: Hotair promotes the migration and proliferation in ovarian cancer by miR-222-3p/CDK19 axis
Source: Cell Mol Life Sci. 2022 Apr 22;79(5):254. doi: 10.1007/s00018-022-04250-0 (PMC9033702; doi:10.1007/s00018-022-04250-0)
Supplement: Supplementary file 1 — Supplementary file1 (DOC 13402 KB) [file 18_2022_4250_MOESM1_ESM.doc]

**Hotair [Promote](C:/Users/lenovo-L/AppData/Local/youdao/dict/Application/8.9.9.0/resultui/html/index.html#/javascript:;)s the Migration and Proliferation in Ovarian Cancer by miR-222-3p/CDK19 Axis**

**Lili Fan^1, 2 +^, Han Lei ^1 +^, Ying Lin^1^, Zhengwei Zhou^1^,** **Juanni Li^1^, Anqi Wu^1^, Guang Shu^1^,**

**Sébastien Roger^3^, Gang Yin^1 *^**

^1^Department of Pathology, Xiangya Hospital, School of Basic Medical Sciences, Central South University, Changsha 410000, Hunan Province, China.

^2^Guangzhou Key Laboratory of Formula-Pattern of Traditional Chinese Medicine, School of Traditional Chinese Medicine, Jinan University, Guangzhou, 510632, Guangdong, People’s Republic of China.

^3^EA4245 Transplantation, Immunologie, Inflammation, University of Tours, 37032, Tours, France.

^*^ Corresponding author: Gang Yin, Ph.D.

Department of Pathology, Xiangya Hospital, School Medical Sciences, Central South University, Changsha 410000, Hunan Province China, [gangyin@csu.edu.cn,](mailto:gangyin@csu.edu.cn,) 13677359115.

**^+^** These authors contributed equally to this work.

**Supplementary Table**

**Supplementary Table S1. Sequences of primers.**

| Items | Sequence |
| --- | --- |
| Hotair qPCR primers | F：AGCCCTAGCCTTTGGAAGCT |
|  | R：ACCCATGTGTCTCAAGATGCATT |
| Hotair CDS primers | F：gactcgcctgtgctctggagcttgatccga |
|  | R：atgcataaaaccaccacacacacacaacc |
| CDK19 qPCR primers | F：GGATTTGTTTGAGTACGAAGGGT |
|  | R：CTACAAGCCGACATGGATATTCC |
| CDK19 CDS primers | F：atggattatgatttcaaggcgaagc |
|  | R：tcagtaccggtgggcctggtgag |
| GAPDH qPCR primers | F：5’-GTCGCCAGCCGAGCCACATC-3’ |
|  | R：5’-CCAGGCGCCCAATACGACCA-3’ |
| U6 | F：CTCGCTTCGGCAGCACATA |
|  | R：AACGATTCACGAATTTGCGT |

Note. F: Forward; R: Reverse.

**Supplementary Materials**

**
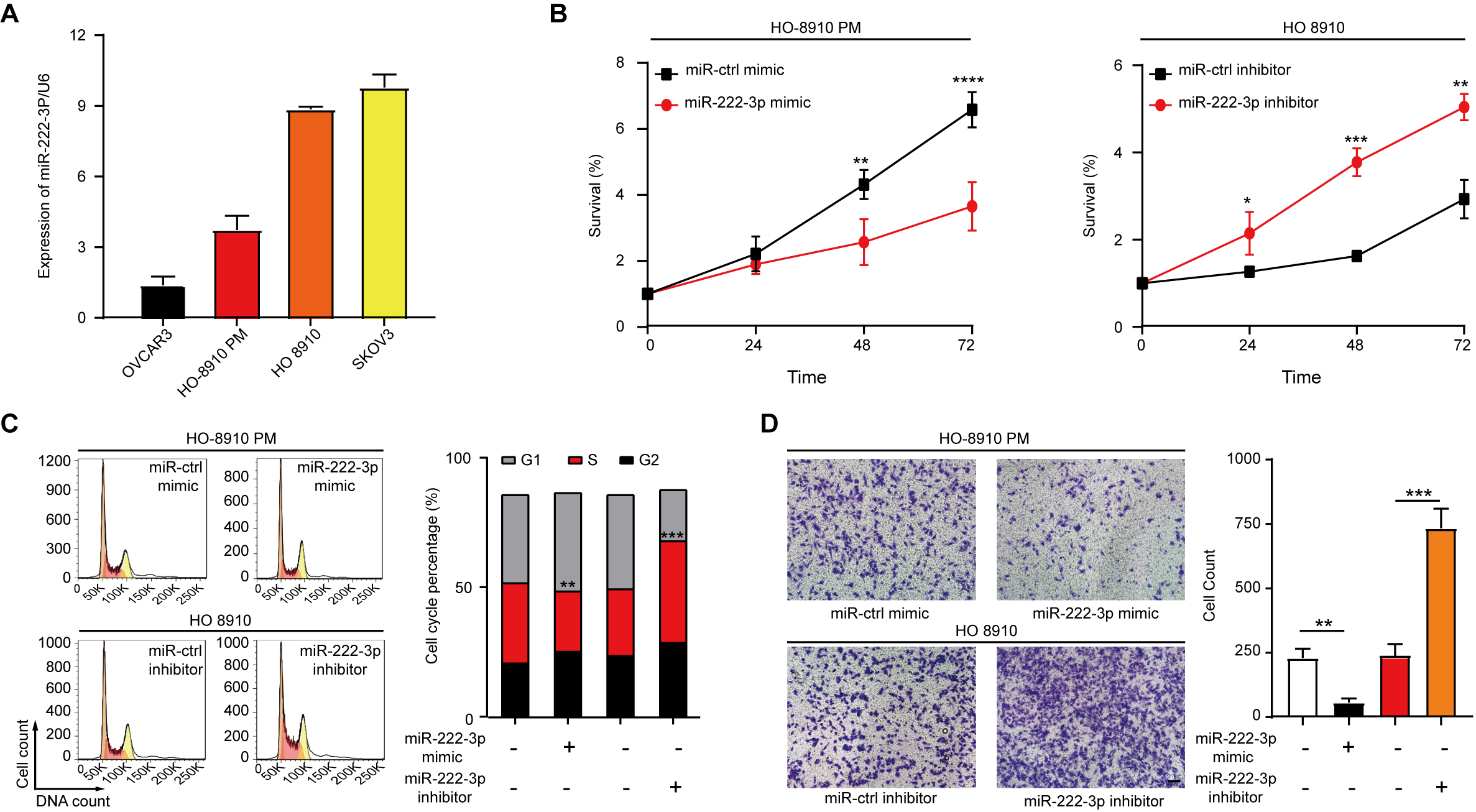
**

**Fig. S1 Expression level** **and function assays of miR-222-3p in OC cells.**

**(A)** Differential relative miRNA expression of miR-222-3p in four OC cell lines. **(B, C)** CCK-8 assays **(B)** and Flow cytometry assays **(C)** revealed that miR-222-3p could affect the proliferation of OC (HO-8910 PM, HO 8910) cells. **(D)** Transwell assays revealed that miR-222-3p could affect the migration of OC cells. 100×microscopic view of the bar, 100 µm. All data **(B-D)** represent the mean ± SD in different assays, revealed by unpaired two-tailed t-test.

**
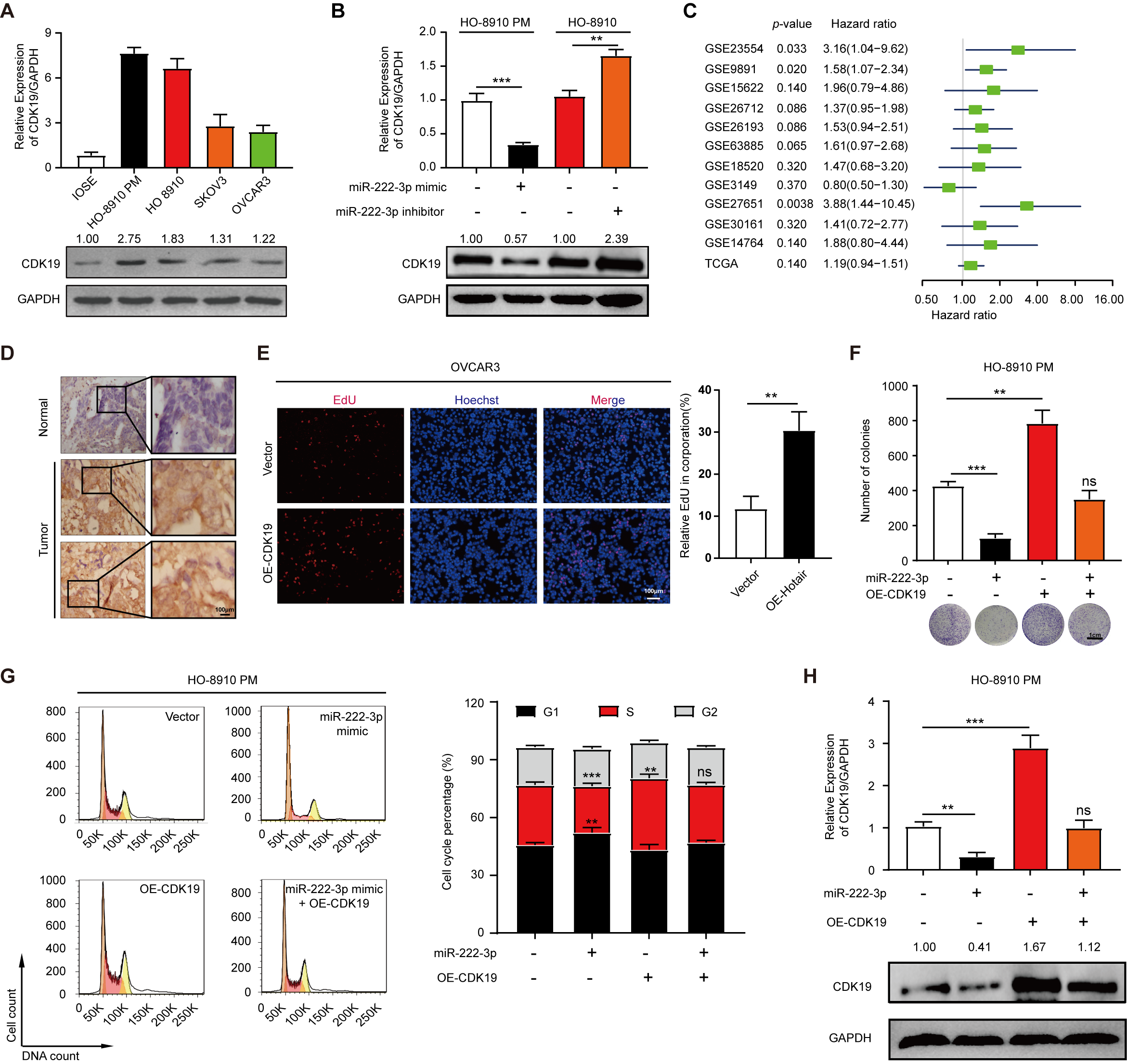
**

**Fig. S2 CDK19 was highly expressed in OC cells and promoted the proliferation of OC cells.**

**(A)** qPCR and Western blotting analyses of CDK19 levels in IOSE and four OC cell lines. **(B)** Western blotting analyses were used to detect CDK19 protein levels after transfection with miR-222-3p mimic or inhibitor in HO-8910 PM cells or HO 8910 cells, respectively. **(C)** Meta-analysis describing forest plots of CDK19 expression as univariate. **(D)** Representative images of CDK19 expression in normal and tumor ovary tissues. Bar, 100 µm (Left) and 30 µm (Right). **(E)** The proliferation ability of OVCAR3 after CDK19 transfection was assessed by the EdU assays, the results were determined by an unpaired two-tailed t-test. **(F, G)** The colony formation assay **(F)** and flow cytometry assay **(G)** revealed inhibition of proliferation when HO-8910 PM cells were transfected with miR-222-3p mimic. Recovery assays showed that miR-222-3p suppressed the proliferation of HO-8910 PM cells due to its inhibitory effect on CDK19. **(H)** qPCR and Western blotting analyses of CDK19 expression levels in HO-8910 PM cells, and Image J calculated the relative expression rate. All data represent the mean ± SD in different assays. The results of **Fig. S2F-H** are presented as mean ± SD and determined by unpaired Two-way ANOVA. **p*<0.05, ***p*<0.01, ****p*<0.001, *****p*<0.0001. All of the experiments were performed in triplicate.

**
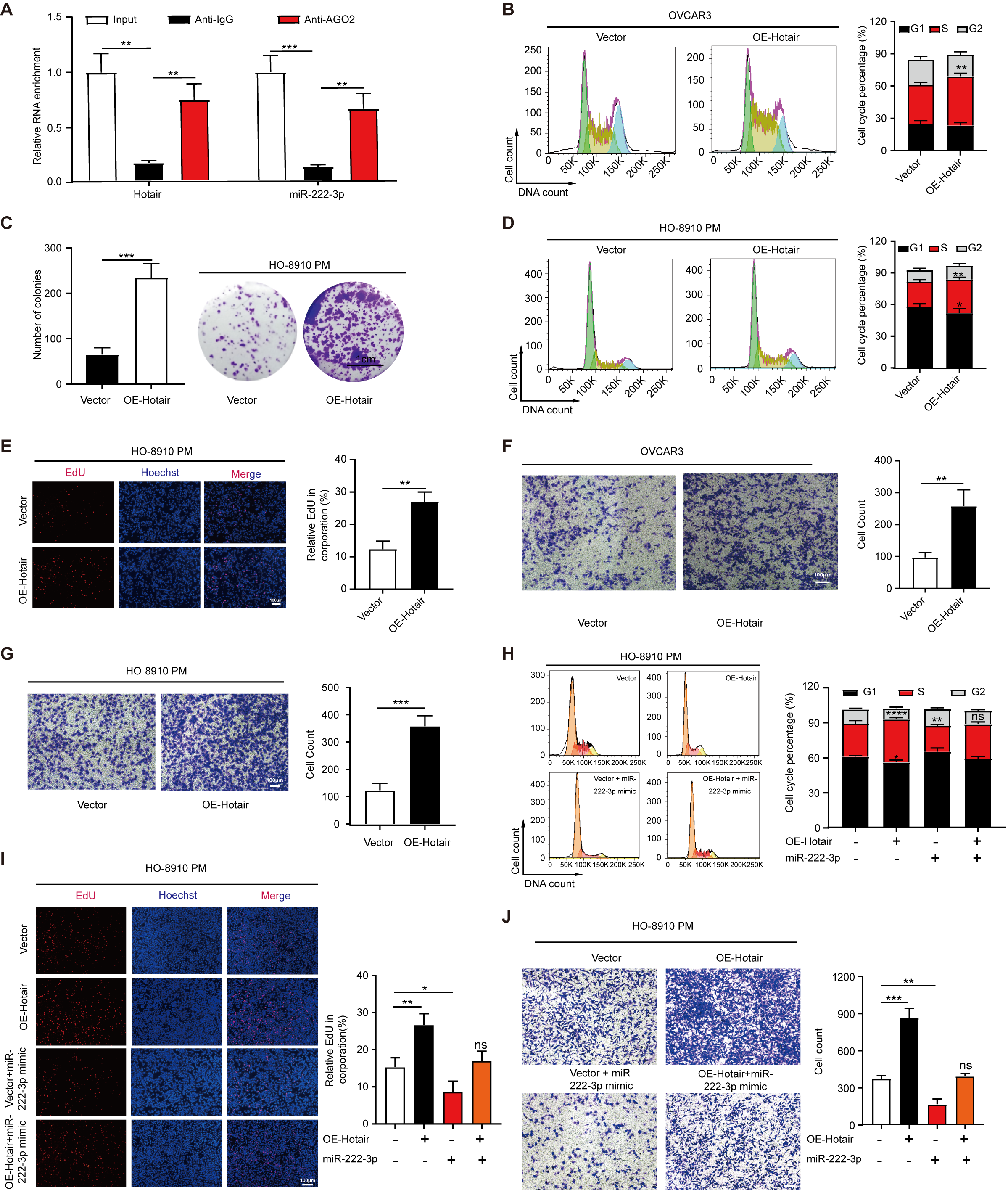
**

**Fig. S3 Hotair can promote the proliferation and migration of OC cells.**

**(A)** Anti-AGO2 RIP was performed in HO 8910 cells, followed by qRT-PCR to detect the expression of LncRNA Hotair or miR-222-3p associated with AGO2. **(B-E)** Colony formation **(C)**, Flow cytometry assay **(B, D)**, and EdU assay **(E)** showed that OC cell proliferation was promoted after transfection with OE-CDK19 plasmids. **(F, G)** Transwell assay showed that OC cells migration was promoted after transfection with OE-CDK19 plasmids. The number of cells was counted by Image J (Right). **(H)** The flow cytometry assay was used to detect the rescue effect of OE-Hotair on OC cells proliferation abilities (Left). Results are presented as mean ± SD and determined by unpaired Two-way ANOVA (Right). **(I)** EdU assay was used to detect the rescue effect of OE-Hotair on OC cells proliferation abilities (Left). Scale bar, 100 µm. The number of cells was counted (Right). **(J)** Transwell assay was used to detect the rescue effect of OE-Hotair on OC cells' migration abilities. Scale bar, 100 µm (Left). The number of cells was counted (Right). Results are presented as mean ± SD and determined by unpaired Two-way ANOVA (Right). **p*<0.05, ***p*<0.01, ****p*<0.001, *****p*<0.0001.
